# Diagnosis and Management of Button Battery Ingestion Complicated by Tracheo-Esophageal and Aorto-Esophageal Fistulas

**DOI:** 10.3390/diagnostics12102369

**Published:** 2022-09-29

**Authors:** Ludovica R. M. Lanzafame, Alfredo Blandino, Giuseppe Cicero, Placido Romeo, Salvatore Agati, Rosanna Zanai, Antonio Celona, Christian Booz, Vitali Koch, Silvio Mazziotti, Tommaso D’Angelo

**Affiliations:** 1 Department of Biomedical Sciences and Morphological and Functional Imaging, University Hospital Messina, 98121 Messina, Italy; 2 Department of Diagnostic and Interventional Radiology, A.O.U. Policlinico San Marco, 95123 Catania, Italy; 3 Pediatric Cardiac Surgery, “Centro Cardiologico Pediatrico del Mediterraneo-Bambino Gesù”, 98039 Taormina, Italy; 4 Pediatric Intensive Care Unit, “Centro Cardiologico Pediatrico del Mediterraneo-Bambino Gesù”, 98039 Taormina, Italy; 5 Department of Radiology, “S. Vincenzo” Hospital Taormina, 98121 Messina, Italy; 6 Division of Experimental Imaging, Department of Diagnostic and Interventional Radiology, University Hospital Frankfurt, 60590 Frankfurt, Germany; 7 Department of Radiology and Nuclear Medicine, Erasmus MC, 3015 Rotterdam, The Netherlands

**Keywords:** foreign bodies, endoscopy, computed tomography, X-ray, esophageal fistula, pediatric emergency medicine

## Abstract

Button battery ingestion (BBI) is common in children and its prevalence has increased in the last decades. BBI can be responsible for very severe and potentially fatal complications if not promptly detected. We describe the successful management of two cases of BBI that occurred in two previously healthy infants. Both patients presented with vague symptoms and no witness of foreign body ingestion. The prolonged time of exposure to the corrosive effects of disk batteries was responsible for the development of tracheo-esophageal fistula (TEF) and aorto-esophageal fistula (AEF). We demonstrate how prompt diagnosis and management are crucial for the infants’ survival.

## 1. Introduction

Foreign body ingestion is common in the pediatric population. Button batteries can be found in many household objects, such as remote controls, games and toys, hearing aids, watches, calculators, and flashlights. The prevalence of button battery ingestion (BBI) has increased during the last decades [1,2]. Despite most the ingested batteries passing through the gastrointestinal system uneventfully, a major complication or death can occur in 12.6% of children younger than 6 years who swallowed batteries with a diameter ≥20 mm. Patients may be initially asymptomatic or present with nonspecific symptoms. Prompt diagnosis and treatment are challenging, considering that most of these events are often unwitnessed [2]. We present two different cases of potentially life-threatening complications of BBI, both successfully managed.

## 2. Case 1

**Figure 1:**
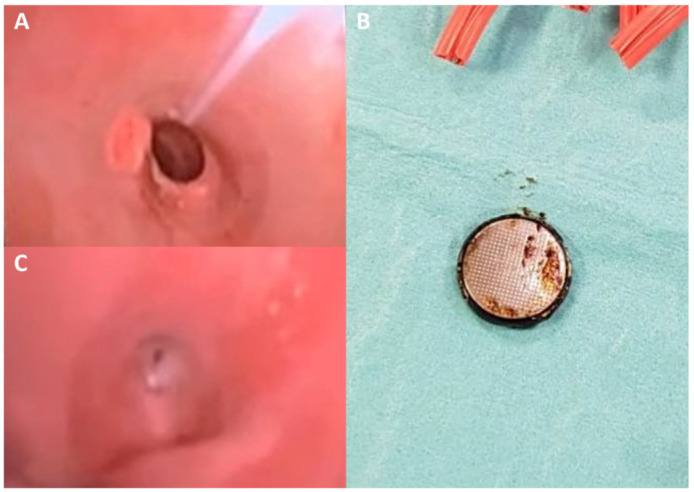
Esophagoscopy image shows the presence of a foreign metallic body (disk battery) in the middle esophagus (**A**), which was promptly removed (**B**). A repeated endoscopy performed 40-days after the first procedure, showed the persistence of TEF (**C**).

A 12-month-old baby male was admitted to the pediatric emergency department with a history of fever, cough, respiratory distress, progressive dysphagia, and sporadic non-bilious vomiting. The patient was febrile (38 °C) at the admission, heart rate of 100/min, and respiratory rate of 30/min. Laboratory tests were normal. Chest X-ray showed the presence of a metallic foreign body projecting adjacent to the trachea and the presence of bilateral pulmonary opacities. Esophagoscopy and bronchoscopy confirmed the presence of a metallic object at the level of the middle esophagus and revealed the presence of a tracheoesophageal fistula (TEF) at the tracheal bifurcation. The patient was successfully managed with endoscopic retrieval of the battery (Figure 1A,B). Subsequently, hospitalization was characterized by recurrent respiratory tract infections caused by Candida albicans and Pseudomonas aeruginosa. Thirty days after the first esophagoscopy, a control endoscopy documented the persistence of the fistula (Figure 1C). The infant underwent complete enteral feeding to allow for complete resolution of lung infection prior to surgical management. After a failed endoscopic attempt, a collegial decision on an open surgical approach was made. The patient underwent median sternotomy with ligation of the fistula and reconstruction of the fistulous pathways. On the fifteenth postoperative day, a radiological upper digestive tract study showed no evidence of contrast media extravasation and successful resolution of the TEF. The infant was discharged twenty-five days after surgery and two months later his condition was stable at ambulatory follow-up.

## 3. Case 2

**Figure 2:**
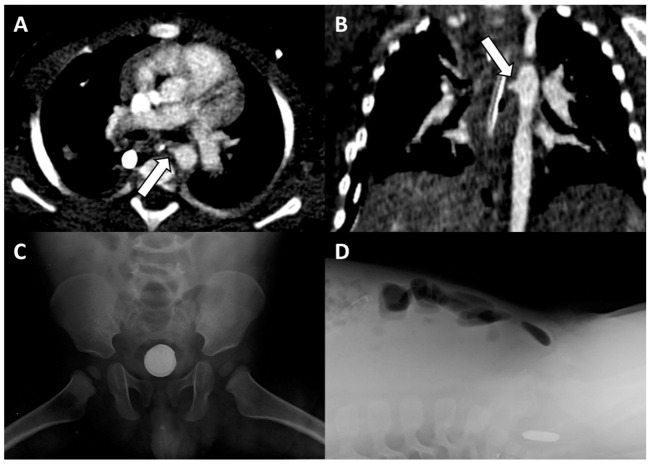
Contrast-enhanced CT angiography images along the axial (**A**) and coronal (**B**) planes, showing the aorto-esophageal fistula (*arrows*). Antero-posterior (**C**) and lateral (**D)** X-rays of the abdomen demonstrate the presence of a metallic foreign body with “double-ring” and “step-off” signs in the pelvic region, suggestive of disk battery in the rectum.

**Figure 3:**
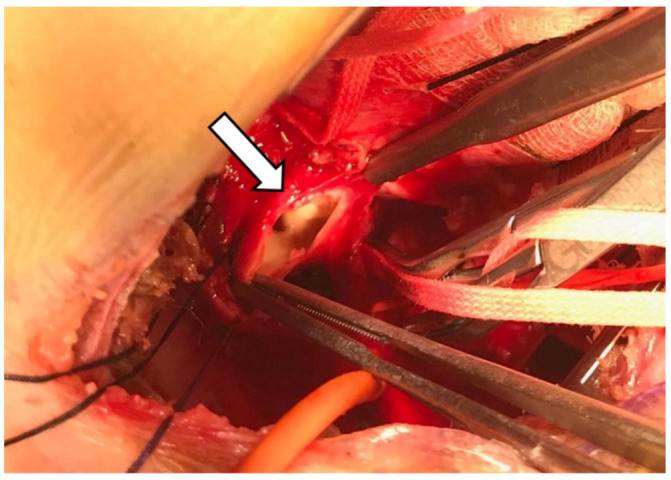
Intraoperative photo revealing the aorto-esophageal fistula (*arrow*).

A 13-month-old male was admitted to the emergency department for hematemesis. Upon arrival, he was unconscious, into cardio-circulatory arrest (blood pressure not detectable, heart rate 145 bpm, respiratory rate 35/min, SpO_2_ 73%) and was promptly resuscitated by the rescuers’ team. Parents referred the baby had been febrile for the previous 9 days with associated dysphagia. A contrast-enhanced CT angiography (CTA) was performed, and it showed contrast medium leakage into the stomach, with the presence of an aorto-esophageal fistula (AEF) at the upper thoracic part of the esophagus (Figure 2A,B). This hypothesis was furtherly supported by the presence of a round metallic body in the lumen of the rectum (Figure 2C,D). Subsequently, the patient underwent aortography that didn’t show any evidence of AEF. On the same day, the disk battery was removed from the rectum. However, the patient underwent an urgent surgical procedure for massive upper gastrointestinal tract bleeding in the suspicion of esophageal lacerations, which were confirmed and promptly clipped. However, a repeated CTA demonstrated the persistence of an aortic penetrating ulcer and the presence of AEF in the upper esophagus. The infant was immediately referred to a pediatric cardiac surgery department and he underwent a left posterolateral thoracotomy. A one-centimeter fistula between the aorta and the esophagus was found and repaired (Figure 3). Follow-up CTA, performed 2 weeks after surgery, confirmed the good resolution of the aortorrhaphy and the patient had an uneventful outcome and was discharged.

## 4. Discussion

The ingestion of foreign bodies is common in children. Button batteries account for 2% of foreign bodies swallowed by children, but according to National Capital Poison Center (NCCP), BBI frequency has increased over the last decades. Up to 2021, 271 severe cases and 69 fatal cases related to BBI have been reported. Among fatal cases, 26 were caused by AEF (37.6%) and 13 by TEF (18.8%) [1,2,3]. BBI-related injury is associated with pressure necrosis and corrosive hydroxyl ions, occurring with the leakage of alkaline contents [4]. Predictive factors for severe complications include the prolonged time of impaction (>2–3 h), battery size (>20 mm), and high voltage (i.e., 3 V) [5].

Most major events are related to unwitnessed ingestion (56.2%) and delayed diagnosis [6]. 

Severe complications usually occur when button batteries remain lodged into the esophagus, a condition that can potentially lead to ulceration, perforation, and fistulization with surrounding tissues. BBI complications include pneumonia, mediastinitis, hemorrhage, esophageal ulceration and perforation, sepsis, tracheoesophageal fistula, and aorto-esophageal fistula [7].

The clinical presentation is often non-specific, and BBI should be considered in the differential diagnosis in previously healthy infants who present with dysphagia, vomiting, fever, cough, or irritability, especially if symptoms have a sudden onset [8]. In both our cases, the little patients presented with vague symptoms, and no witness or suspicion of foreign body ingestion was reported.

Chest X-ray represents the first diagnostic tool to attest the presence of a foreign body, its location, size, and shape, and it may help distinguish disk batteries from other metallic objects (i.e., “double halo” or “double ring” sign on antero-posterior view and the “step-off” sign on lateral view) [9].

Both the North American Society for Pediatric Gastroenterology, Hepatology and Nutrition (NASPHGAN) and the European Society for Paediatric Gastroenterology Hepatology and Nutrition (ESPHGAN) recommend immediate endoscopic retrieval when the button battery is detected in the esophagus [4,9]. 

However, our first case demonstrated that a tracheoesophageal fistula was present despite the prompt endoscopic retrieval, probably due to prolonged contact duration between the battery and the esophageal wall. TEF mortality related to BBI accounts for 11.4% and the management options include a conservative approach, to allow for spontaneous TEF closure or surgical repair. Despite no recommendations or guidelines are currently available, some authors have suggested that a conservative approach is favorable when a patient’s clinical condition is stable, in order to minimize surgery-related complications and recurrences. Moreover, they suggested that a conservative approach should last at least 8 weeks prior to opting for surgical repair. However, a prolonged watchful waiting strategy in these patients may increase the risk for reflux aspiration and respiratory distress [10]. The choice of the proper surgical technique can also be challenging and is influenced by several factors including the location, the size, and contour of the TEF, and the presence of esophageal, bronchopulmonary, or systemic disease. Single-stage primary repair of both the trachea and esophagus is the treatment of choice in case of small lesions (<5 mm). Large fistulas (≥10 mm) may require concomitant tracheal resection and reconstruction for the repair of the TEF [11,12].

Once the disk battery passes into the stomach or bowel, the transit is generally without complications, but patients still need clinical monitoring and repeated X-rays to check for the battery progression [13].

However, the finding of a disk battery in the stomach or bowel should not let the guard down against the presence of BBI-related complications. In fact, our second case presented with hematemesis, melena, hemodynamic instability, and the presence of AEF was reported when the disk battery was lodged in the colon [14].

In the presence of such symptoms, a contrast-enhanced CT scan should be timely performed in order to assess the presence of this rare and life-threatening complication and to transfer the patient to a cath-lab or operation room [4,13,15]. 

In our case, first-line aortic angiography failed to detect the presence of the fistula, probably due to compression made by a massive clot surrounding the nasogastric tube at the level of AEF. This case further emphasizes the importance of CTA for pre-operative assessment of AEF, since it allowed for the extraluminal assessment of the aorta and to identify the location and the size of the fistula.

AEF is a very rare and generally fatal BBI-related complication. According to scientific literature, only nine other cases have been diagnosed and successfully managed, with the survival of the patient (Table 1). In all cases, patients presented with hematemesis, and BBI was unwitnessed in most cases. The diagnosis was made by CTA in six cases, angiogram in two cases, and during the operation in one patient. AEF was surgically repaired in seven patients and treated by endovascular approach in three cases [7,16,17,18,19,20,21,22,23].

Clinicians should be aware of severe complications that derive from BBI even days or weeks after button battery removal, due to persistent alkaline-induced liquefactive necrosis. A high level of guarding should be kept in presence of a battery with a diameter >20mm (3V), with prolonged time of impaction, and in patients younger than five years old [1].

In conclusion, BBI can lead to life-threatening complications and diagnosis can often be challenging due to unwitnessed or nonspecific clinical presentation. 

In presence of sudden onset symptoms in previously healthy little patients, the possibility of foreign body ingestion and BBI should always be considered. Radiological examinations, including X-rays and CT scans, followed by endoscopy are necessary, either to confirm the presence of the foreign body or to assess the presence of complications. Aorto-esophageal fistula and trachea-esophageal fistula are two potentially fatal events related to BBI that should always be carefully excluded. We demonstrated how important early diagnosis and prompt treatment were for patients’ survival.

## Figures and Tables

**Table 1:** Survival cases of BBI complicated by aorto-esophageal fistula.

	Sex	Age (Years)	Battery Size	Length of BB Exposure	Clinical Presentation	Diagnosis	Management
Spiers (2012) [16]	M	1	20 mm	14 h	Hematemesis	CTA	Surgical repair
Granata (2018) [17]	F	2	Unknown	Unknown	Hematemesis and hemodynamic shock	Angiogram	Endovascular stent
Mahajan (2019) [18]	F	3	Unknown	Unknown	Hematemesis	CTA	Surgical repair
Bartkevics (2020) [19]	F	1	20 mm	Unknown	Hematemesis and melena	CTA	Surgical repair
Sinclair (2021) [20]	F	6	21 mm	6 h	Hematemesis	Angiogram	Endovascular stent
Wakimoto (2021) [21]	F	1.5	23.5 mm	Unknown	Hematemesis	CTA	Surgical repair
Alreheili (2021) [7]	M	2.5	20 mm	16 h	Hematemesis, melena and nasal bleeding	CTA	Vascular plug device
Gibbs (2021) [22]	F	1.5	20 mm	Unknown	Hematemesis	CTA	Surgical repair
Muhieldin (2022) [23]	F	1.5	21.6 mm	Unknown	Hematemesis	During operation	Surgical repair
Current Case	M	1	20 mm	Unknown	Hematemesis and cardio-circulatory arrest	CTA	Surgical repair
